# Case report: Abolishing primary resistance to PD-1 blockade by short-term treatment of lenvatinib in a patient with advanced metastatic renal cell carcinoma

**DOI:** 10.3389/fimmu.2023.1115691

**Published:** 2023-01-20

**Authors:** Tingting Tan, Xiaotong Lin, Jing Ling, Rong Wang, Yue Chen, Longmei Cai, Jingyuan Sun, Dehua Wu, Guozhu Xie

**Affiliations:** ^1^ Department of Radiation Oncology, Nanfang Hospital, Southern Medical University, Guangzhou, Guangdong, China; ^2^ The First School of Clinical Medicine, Southern Medical University, Guangzhou, China

**Keywords:** primary resistance, PD-1, lenvatinib, renal cell carcinoma, pembrolizumab

## Abstract

Anti-PD-1 immunotherapy has been extensively used in treatment of patients with advanced metastatic renal cell carcinoma (mRCC). Several prospective clinical trials showed that the combined treatment of anti-PD-1 antibody plus lenvatinib, a potent receptor tyrosine kinase inhibitor (TKI), exhibited high response rate compared with single-agent sunitinib. However, whether the patients with primary resistance to PD-1 blockade could benefit from the addition of lenvatinib is still unclear. Herein, we reported a patient with mRCC who was primary resistant to pembrolizumab and achieved a durable complete response after a short-term treatment with lenvatinib. This case report indicates that the patients with primary resistance to anti-PD-1 therapy could benefit from the short-term lenvatinib in combination with anti-PD-1 therapy, and provides a useful paradigm worthy of establishing a clinical trial for mRCC patients with primary resistance to anti-PD-1 therapy.

## 1 Introduction

Renal cell carcinoma (RCC) is a rapidly progressing malignant tumor of urinary system. Approximately one-third of patients with renal cell carcinoma have metastatic disease at initial presentation (1). Metastatic RCC (mRCC) has been notoriously resistant to conventional radiotherapy and chemotherapy. Currently, immune-checkpoint blockade (ICB) has been extensively used in treatment of patients with advanced mRCC. Recent studies showed that the ICB-based combination treatment had better outcomes than sunitinib for patients with mRCC (2–6). Data from a prospective trial (NCT02853331) showed that the treatment with pembrolizumab plus axitinib resulted in 59.3% of overall objective response rate (ORR) among patients with previously untreated advanced mRCC (4). Another encouraging prospective trial (NCT02811861) showed 71.0% of ORR to the treatment with lenvatinib plus pembrolizumab for mRCC patients without previous systemic therapy (6). Notably, the combination of lenvatinib plus pembrolizumab even showed antitumor activity for patients with previously treated metastatic renal cell carcinoma (7). However, whether the patients with primary resistance to PD1 blockade could benefit from the addition of lenvatinib is still unclear.

Lenvatinib is a novel multitargeted receptor tyrosine kinase inhibitor (TKI) with activity against with VEGFR1-3, FGFR1-4, PDGFRα, RET and KIT proto-oncogenes (8). Compared with other TKIs, lenvatinib has the more potent activity against FGFR1-4 (9, 10), and has shown the higher efficacy as monotherapies than everolimus for RCC (11). However, high incidence rate of grade 3 and 4 adverse events was also observed in lenvatinib group compared with everolimus, which often led to treatment interruption of lenvatinib (11).

Previous studies in mouse tumor isograft models have demonstrated the immunomodulatory activity of lenvatinib in tumor microenvironment, including reducing tumor-associated immunosuppressive macrophages, increasing cytotoxic T cells, and activating interferon γ signaling (12, 13). In the human body, the immune system is able to recognize tumor antigens as not self and mounts an immune response against tumor cells. Once the immune response against tumor is primed in the body, it could be durable (14, 15). Given the potential role of lenvatinib in priming an antitumor immune response, there exists a possibility that just short-term use of lenvatinib could affect primary resistance of tumor to PD-1 blockade. Herein, we reported a patient who was primary resistant to pembrolizumab and achieved a durable complete response after a short-term treatment with lenvatinib.

## 2 Case presentation

A male patient received a diagnosis of renal cell carcinoma in December 2008 at 46 years of age. A computed tomography (CT) scan of the chest and abdomen revealed a left renal tumor with a 4.5cm maximum diameter which was confined to the renal capsule, and he underwent radical nephrectomy on December, 2008. The pathological examination showed a moderately to poorly differentiated renal clear cell carcinoma. All indications suggested a diagnosis of stage I RCC (T1bN0M0) according to the American Joint Committee on Cancer (AJCC) Cancer Staging Handbook, 6th edition. There was no residual tumor after surgery at the primary site, and the lymph nodes removed were not found to be involved. The patient underwent periodical CT examination after surgery.

**Figure 1:**
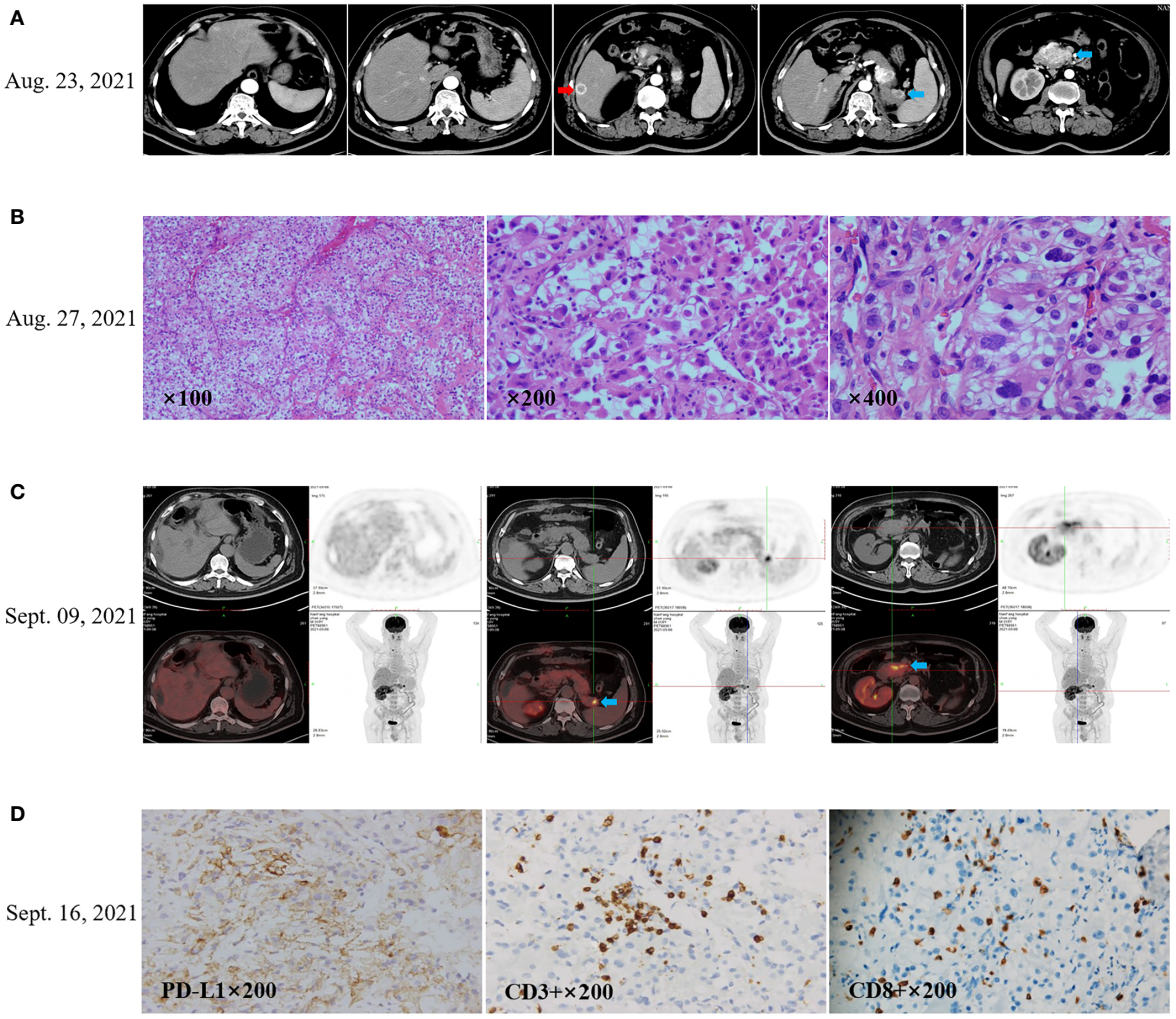
Recurrence after radical left nephrectomy of the patient’s with renal clear cell carcinoma. **(A)** CT scan (August 23, 2021) showed the first recurrence after nephrectomy in 13 years. The red arrow indicates the prominent lesion in the liver, and the blue arrow indicates the pancreatic lesion. **(B)** Laparoscopic partial hepatectomy was performed for the largest intrahepatic lesions on August 26, 2021, and postoperative pathology confirmed liver metastasis of renal clear cell carcinoma. **(C)** The images of positron emission tomography CT (PET-CT) on September 09, 2021 showed hypermetabolic pancreatic lesions, no hypermetabolism in the residual liver, and no additional hypermetabolic lesions. **(D)** Immunohistochemistry showed that PD-L1 expression in the tumor tissue was 40% for tumor proportion score (TPS), CD3^+^ TIL density was 100 cells/HPF, and CD8^+^ TIL density was 50 cells/HPF.

The patient remained disease-free until contrast enhancement CT scan of upper abdomen revealed several new lesions at pancreas and liver on August 23, 2021 (**Figure 1A**), due to upper abdominal pain. Laparoscopic partial hepatectomy was performed for the largest intrahepatic lesions on August 26, 2021, and postoperative pathology confirmed liver metastasis of renal clear cell carcinoma (**Figure 1B**). The images of positron emission tomography CT (PET-CT) on September 09, 2021 showed three hypermetabolic lesions in pancreas and no hypermetabolism in the residual liver and other sites (**Figure 1C**). The pancreaticobiliary duct was seriously obstructed due to the compression by the metastatic tumor, a stent was implanted by endoscopic retrograde cholangio-pancreatography (ERCP) on September 17, 2021.

Immunohistochemical staining showed the tumor proportion score (TPS) of PD-L1 expression was 40% according to Allred criteria using the DAKO PD-L1 22C3 PharmDx assay (Agilent Technologies; Carpinteria, CA, USA) (**Figure 1D**). Since September 22, 2021, the patient received anti-PD-1 treatment with pembrolizumab (200 mg intravenously once every 3 weeks). The patient received a palliative radiotherapy to the pancreatic lesions on September 27, 2021. with a total of 40 Gy in five fractions once every other day using 6-MV photons by means of a coplanar nine-field intensity-modulated image-guided technique (IMRT).

**Figure 2:**
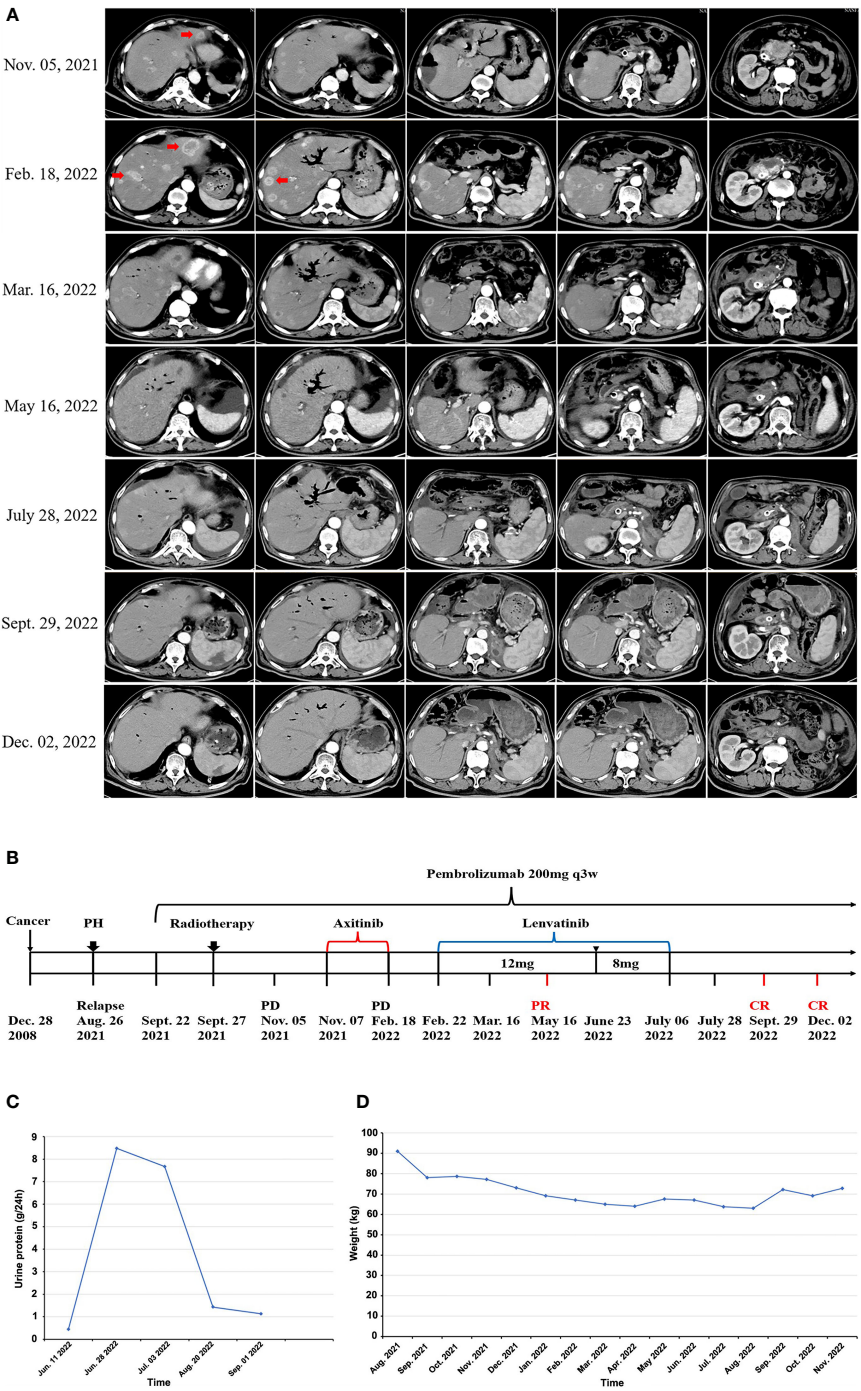
Short-term lenvatinib overcomes primary resistance of mRCC to anti-PD1 therapy. **(A)** After 3 cycles of pembrolizumab monotherapy, CT scan on November 05, 2021 showed disease progression, the patient was still resistant to the combination of axitinib with pembrolizumab, with significantly enlarged and increased number of metastases in liver (Feb 18, 2022), the addition of lenvatinib to pembrolizumab led to rapid tumor regression shown in a follow-up CT scan on March 16, 2022 (23 days after lenvatinib initiation), CT examination in May 2022 revealed almost complete response (CR) of the intrahepatic and pancreatic lesions, all lesions continued to regress in the two CT scans after lenvatinib was discontinued, with a sustained complete response as shown in the CT scan image on September 29, 2022 and December 02, 2022. **(B)** Diagram of the patient’s entire treatment process, PH representing laparoscopic partial hepatectomy (August 26, 2021), representing lenvatinib dose reduction due to AEs (June 23, 2022). **(C)** After 1 month of suspension of the use of lenvatinib, the patient’s 24-hour urine protein test showed that urine protein gradually returned to normal. **(D)** After discontinuation of lenvatinib, the patient gradually gained weight.

After 3 cycles of pembrolizumab, a surveillance CT scan on November 05, 2021 showed disease progression, with several new metastases in liver (**Figure 2A**). The patient thus received the combined treatment with pembrolizumab (200 mg q3W) plus axitinib (5 mg q12h) from November 07, 2021. After 3-cycle treatment with pembrolizumab plus axitinib, a surveillance CT scan was performed on February 18, 2022, and showed further disease progression with significantly enlarged and increased number of metastases in liver compared with that on November 05, 2021 (**Figure 2A**). On February 22, 2022, axitinib was replaced by lenvatinib (12mg orally once daily) and pembrolizumab treatment was continuously administered as before. A follow-up CT scan in May 16, 2022 (3 months after lenvatinib initiation) showed the metastatic lesions in the liver had significantly decreased in size (**Figure 2A**). But at the same time, the patient experienced grade 3 adverse events (AEs) of proteinuria and hypertension according to the standard CTCAE5.0 criteria, so the dose of lenvatinib was reduced to 8mg once daily on June 23, 2022. Lenvatinib had to be discontinued due to aggravation of proteinuria on July 6, 2022, and pembrolizumab monotherapy was maintained. The whole process of treatment was shown in **Figure 2B**.

## 3 Results

Immunohistochemistry (IHC) showed the tumor proportion score (TPS) of PD-L1 expression was 40%, indicating potential response to anti-PD-1 therapy. However, for this patient, tumors did not respond to anti-PD-1 pembrolizumab monotherapy for 1.5 months, and disease continuously progressed even though axitinib was added to pembrolizumab for later 3.5 months (**Figure 2A**). After that, lenvatinib instead of axitinib was added to the treatment of pembrolizumab. Remarkably, an abdomen CT scan showed a dramatic treatment response of the patient’s known lesions at 23 days after addition of lenvatinib to pembrolizumab (**Figure 2A**). All lesions had significantly regressed at next follow-up CT scan at 3 months after starting lenvatinib (May 16, 2022). Due to proteinuria and hypertension, the dose of lenvatinib was reduced to 8mg once daily on June 23, 2022. Lenvatinib was eventually discontinued on July 6, 2022, and pembrolizumab monotherapy was maintained after that. Nevertheless, all lesions had continuously regressed at multiple follow-up CT scans, and achieved complete response as shown by the CT scan images on September 29, 2022, and a sustained complete response was observed in a recent CT scan images on December 2, 2022 (**Figure 2A**).

The patient experienced severe proteinuria (grade 3) and hypertension (grade 2) during lenvatinib treatment, and lenvatinib had to be discontinued on July 6, 2022. After 1 month of suspension of the use of lenvatinib, the patient’s 24-hour urine protein test showed that urine protein gradually returned to normal (**Figure 2C**), hypertension symptom disappeared, and the patient no longer needed to take antihypertensive drugs. Additionally, ascites, bloating, diarrhea, fatigue, edema, and other lenvatinib-related adverse events were relieved. The body weight of patient was significantly increased (**Figure 2D**) and ECOG performance status was decreased from 3 points to 1 point, indicating the quality of life was significantly improved.

## 4 Discussion

ICB immunotherapy has become an important approach in the treatment of advanced cancers. However, the proportion of patients who can benefit from such therapy remains limited, with an estimated average response rate of 25% for patients with solid tumors, owing to primary or acquired resistance (16). Recently, Tae K. et al. proposed four distinct types of human cancer based on the presence of PD-L1 expression and tumor-infiltrating lymphocytes (TILs) in the tumor immune microenvironment (TIME), to better identify tumor response or resistance to anti-PD therapy. According to this classification, tumors could be divided into four types: PD-L1−/TIL− (type I); PD-L1+/TIL+ (type II); PD-L1−/TIL+ (type III); and PD-L1+/TIL− (type IV) (16). Primary resistance to anti-PD therapy was defined as the failure to initially respond to anti-PD therapy in the type II tumor, which has both PD-L1 expression and T cell infiltration (16). For this case, TPS of PD-L1 expression in tumor was 40%, and CD3+ TIL density was 100 cells/HPF (CD8+ TIL density was 50 cells/HPF), thus indicating a type II tumor with primary resistance to anti-PD therapy, which was evidenced by the fact that the tumor continuously progressed during pembrolizumab treatment for 5 months.

Multiple approaches are being developed to improve the response to anti-PD-1 therapy, including the combination treatment with other therapies. Combination with TKI targeted therapy has proved effective for RCC in several clinical trials. Pembrolizumab combined with axitinib was approved by the FDA in advanced RCC after the KEYNOTE-426 trial found that the combination was superior to monotherapy with sunitinib (4). However, for this case, the patient presented primary resistance to pembrolizumab monotherapy and even in combination with axitinib. Lenvatinib is a novel potent multitarget TKI that performs its action through the inhibition of VEGFR 1-3, FGFR 1-4, PDGFRα, RET and KIT (8). Lenvatinib plus pembrolizumab demonstrated a promising antitumor activity in mRCC patients (6). However, grade 3 or higher adverse events occurred in up to 82.4% of the patients who received lenvatinib plus pembrolizumab. Notably, preclinical studies demonstrated that lenvatinib pretreatment could induce an immune-activating tumor microenvironment, resulting in significantly greater antitumor activity compared with anti-PD-1 treatment alone (12, 13), indicating a possibility that just short-term use of lenvatinib could initiate a potent and durable antitumor response to PD-1 blockade in patients with a type II tumor that is originally resistant to anti-PD therapy. Herein, we reported, for the first time, that a patient who was primary resistant to pembrolizumab plus axitinib, achieved a lasting response after a short-term treatment with lenvatinib. The miraculous effect observed herein provided further evidence for lenvatinib of modulating the tumor immune microenvironment and overcoming primary resistance of tumor to ICB.

For this case, radiotherapy was another factor that should be considered for contributing to the effect of anti-PD-1 therapy. Several cases had been reported that local radiotherapy, especially SBRT, could produce an abscopal effect by activation of the immune system. There are several prospective clinical trials investigating the abscopal effect (17–19). However, consistent results regarding occurrence of abscopal effect are highly variable. In general, these trials exploring expansion of abscopal effect still only provide relatively low occurrence rate of abscopal activity (19). For this case, a palliative radiotherapy (40Gy/8F) was delivered to metastatic lesions in pancreas. Unfortunately, disease, except for lesions in the irradiation field, continuously progressed during 5 months after radiotherapy until the treatment with lenvatinib. Therefore, tumor regression is unlikely to be an abscopal effect induced by radiotherapy. Additionally, the limitation of this case report is that it was not clear which markers were associated with the patient’s durable response and what was the specific mechanism by which lenvatinib overcame primary resistance to perbrolizumab in this patient. It would be worthwhile to design a new clinical trial and a rigorous experiment to demonstrate.

## 5 Conclusion

This patient’s surprising systemic response after short-term lenvatinib in combination with pembrolizumab provides new insights into the role of short-term lenvatinib in overcoming primary resistance of tumor to anti-PD-1 therapy.

## Data availability statement

The original contributions presented in the study are included in the article/supplementary material. Further inquiries can be directed to the corresponding authors.

## Ethics statement

The studies involving human participants were reviewed and approved by The Ethics Committee of the Nanfang Hospital. The patients/participants provided their written informed consent to participate in this study. Written informed consent was obtained from the individual(s) for the publication of any potentially identifiable images or data included in this article.

## Author contributions

TT, XL, and JL designed the study and drafted the entire manuscript. RW and YC are responsible for patient information acquisition and postoperative follow-up. LC and JS are responsible for imaging and image processing. DW and GX conceived and designed the study, and guided the article revision. All authors contributed to the article and approved the submitted version.
